# Supplementary material for: Evolutionary age correlates with range size across plants and animals
Source: Nat Commun. 2025 Aug 23;16:7894. doi: 10.1038/s41467-025-62124-y (PMC12375016; doi:10.1038/s41467-025-62124-y)
Supplement: Supplementary file 1 — Supplementary information [file 41467_2025_62124_MOESM1_ESM.pdf]

Supplementary Table 1

A non-exhaustive compilation of studies examining the relationship between evolutionary age and range size. For each taxonomic group, we indicated in parentheses whether it is based on fossil data, and provided more precise information on the studied group. We included information on the study location and the number of species (or another taxonomic unit) studied. Finally, we indicated the reported relationship between evolutionary age and range size.

Taxonomic group	Location	Taxonomic units	Effect of evolutionary age on range size	Reference
Gastropods and bivalve molluscs (fossils)	North America	408-540	Positive	Jablonski 1987
Plants		21-24 genera	Positive	Ricklefs & Latham 1992
Trilobites, articulate brachiopods, molluscs (fossils)	Worldwide	974 genera	Positive	Miller 1997
Ice-age large mammals (fossils)	Italy	19 species	Linear and peaked	Raia et al. 2006
Molluscs (fossils)	worldwide	97 genera	Mixed	Foote et al. 2007
Plants (Proteacea)	Fynbos Biome	37 species	Neutral	Schurr et al. 2007
Plants (Piperaceae)	Neotropics	67 species	Positive	Paul & Tonsor, 2008
Plants (Rubiaceae)	Neotropics	65 species	Positive	Paul et al. 2009
Beetles (aquatic)	Western Palaearctic	10 lineages (9-30 taxa)	9 neutral, 1 positive	Abellan & Ribera 2011
Damselflies (*Coenagrion*)	Worldwide	16 species	Neutral	Swaegers et al. 2014
Birds	New World	117 clades	Neutral with mean clade range size<br>Positive with total clade range<br>Neutral with tribe mean range size or tribe total range size	Gaston & Blackburn 1997
Birds (*Phylloscopus* warblers)	Old world	42 taxa	Neutral	Price et al. 1997
Birds			Mixed	Webb & Gaston 2000
Birds (*Sylvia* warblers)	Old world	26 species	Positive	Böhning-Gaese et al. 2006
Frogs (Mantellidae)	Madagascar	53 pairs of sister species	Neutral, positive trend	Wollenberg et al. 2011
Bats (Phyllostomidae)	South America	49 species	Positive	Weber et al. 2014
Mammals	Worldwide	222-226 species	Negative	Jones et al. 2005
Fish (*Cyprinella*)	North America	27 species	Positive	Taylor & Gotelli 1994

Reef fishes	Worldwide	183 species in 7 families	Neutral for any of the 7 examined families	Mora et al. 2012
Reef fishes	Worldwide	293 species	Positive	Hodge & Bellwood, 2015
Snakes	Worldwide	536 species	Neutral	Böhm et al. 2017
Birds (Campephagidae)	Indo-Pacific Islands	64 species	Negative	Pepke et al. 2019
Plants	Atlantic Forest	13,283 species	Neutral	Leão et al. 2020
Mammals, reptiles, amphibians, birds	Worldwide	24,236 species	Positive	Guo et al. 2024

Supplementary Table 2

Relationship between evolutionary age and range size. Summary of the Linear Mixed Model for the relationship between evolutionary age and range size for all taxa (*n* = 26,347 species) combined. To test the overall effect of evolutionary age, we included families (*n* = 535) nested within orders *(n* = 106) nested within taxonomic groups (*n* = 7) as random effects. Additionally, we ran similar models for each taxonomic group, but with different random effect structures. For amphibians (*n* = 4,190), birds (*n* = 8,785), reef fishes (*n* = 1,479), terrestrial mammals (*n* = 5,142), and marine mammals (*n* = 70), we ran linear mixed models with family nested within order as random effects. We only included family as a random effect for reptiles (*n* = 5,482) as this only includes the order Squamata. We ran a Linear model without random effects for palms (*n* = 1,199), which only includes the family Arecaceae. SE = standard deviation, df = degrees of freedom, *t* = *t* value, *p* = *p*-value, R^2m = marginal R^2, R^2c = conditional R^2.

Model		Estimate	SE	df	*t*	*p*	R^2m	R^2c
**All taxa**	Intercept	0.15	0.15	5.82	0.98	0.36		
Age + (1 | Groups/Order/Family)	**Age**	**0.16**	**0.00**	**25390.00**	**25.50**	**0.00**	0.02	0.55
**Amphibians**	Intercept	0.01	0.07	0.83	0.18	0.89		
Age + (1 | Order/Family)	**Age**	**0.15**	**0.02**	**4175.00**	**9.41**	**0.00**	0.02	0.20
**Birds**	Intercept	0.08	0.03	25.14	2.43	0.02		
Age + (1 | Order/Family)	**Age**	**0.15**	**0.01**	**8325.00**	**13.45**	**0.00**	0.02	0.20
**Reef fishes**	Intercept	0.06	0.03	31.09	1.72	0.10		
Age + (1 | Order/Family)	**Age**	**0.15**	**0.03**	**1388.00**	**5.93**	**0.00**	0.02	0.15
**Terrestrial mammals**	Intercept	0.03	0.05	20.50	0.62	0.54		
Age + (1 | Order/Family)	**Age**	**0.19**	**0.01**	**5086.00**	**13.27**	**0.00**	0.03	0.27
**Marine mammals**	Intercept	-0.08	0.12	7.25	-0.69	0.51		
Age + (1 | Order/Family)	Age	0.10	0.13	67.89	0.74	0.47	0.01	0.30
**Palms**	Intercept	-0.00	0.01		0.00	1.00		
Age	**Age**	**0.20**	**0.03**		**6.98**	**0.00**	0.04	0.04
**Reptiles**	Intercept	0.03	0.03	34.71	0.91	0.37		
Age + (1 | Family)	**Age**	**0.19**	**0.01**	**5357.00**	**13.06**	**0.00**	0.03	0.17

Supplementary Table 3

Relationship between evolutionary age and range size, excluding outliers. Summary of the Linear Mixed Model for the relationship between evolutionary age and range size for all taxa (*n* = 25,959) combined, excluding very old species with evolutionary age values greater or less than three standard deviations from the mean. As random effects, we included families nested within orders nested within taxonomic groups. Additionally, we ran similar models for each taxonomic group, but with different random effect structures. For amphibians (*n* = 4,120), birds (*n* = 8,690), reef fishes (*n* = 1,457), marine mammals (*n* = 69) and terrestrial mammals (*n* = 5,056), we ran Linear Mixed Models with family nested within order as random effects. We only included family as a random effect for reptiles (*n* = 5,389) as this only includes the order Squamata. We ran a Linear model without random effects for palms (*n* = 1,178), which includes a single family (Arecaceae). SE = standard deviation, df = degrees of freedom, *t* = *t* value, *p* = *p*-value, R^2m = marginal R^2, R^2c = conditional R^2.

Model		Estimate	SE	df	*t*	*p*	R^2m	R^2c
**All taxa**	Intercept	0.15	0.16	5.82	1.00	0.36		
Age + (1 | Groups/Order/Family)	**Age**	**0.16**	**0.07**	**25300.00**	**24.85**	**0.00**	0.02	0.55
**Amphibians**	Intercept	0.02	0.07	0.62	0.28	0.85		
Age + (1 | Order/Family)	**Age**	**0.15**	**0.02**	**4112.00**	**9.60**	**0.00**	0.02	0.19
**Birds**	Intercept	0.09	0.04	23.65	2.45	0.02		
Age + (1 | Order/Family)	**Age**	**0.15**	**0.01**	**8322.00**	**13.12**	**0.00**	0.02	0.20
**Reef fishes**	Intercept	0.06	0.03	30.12	1.81	0.08		
Age + (1 | Order/Family)	**Age**	**0.15**	**0.03**	**1379.00**	**5.78**	**0.00**	0.02	0.16
**Terrestrial mammals**	Intercept	0.03	0.05	18.66	0.67	0.51		
Age + (1 | Order/Family)	**Age**	**0.18**	**0.01**	**5040.00**	**12.93**	**0.00**	0.03	0.27
**Marine mammals**	Intercept	-0.08	0.12	7.10	-0.65	0.53		
Age + (1 | Order/Family)	Age	0.08	0.13	66.17	0.63	0.53	0.01	0.29
**Palms**	Intercept	0.00	0.01		0.00	1.00		
Age	**Age**	**0.19**	**0.03**		**6.64**	**0.00**	0.04	0.04
**Reptiles**	Intercept	0.04	0.03	32.16	1.02	0.32		
Age + (1 | Family)	**Age**	**0.19**	**0.01**	**5312.00**	**12.61**	**0.00**	0.03	0.17

Supplementary Table 4

Relationship between adjusted evolutionary age and range size. Summary of the Linear Mixed Model for the relationship between (adjusted) evolutionary age and range size for all taxa (*n* = 26,347 species) combined. Following Calderon del Cid et al. (2024), we applied the age correction, which uses the speciation and extinction rates to correct species ages for potential 'hidden' speciation events with complete tree sampling. The original study uses true extinction and speciation rate values to simulate the phylogenetic trees; we instead calculated speciation and extinction probabilities by fitting a birth-death model to phylogenetic data, providing maximum likelihood estimates of these rates using the 'bd_ML' function from the 'DDD' R package (Etienne & Haegeman 2023). This adjustment could not be applied to branch lengths smaller than 0.01 Myr. For all species combined, we included families nested within orders nested within taxonomic groups as random effects. Additionally, we ran similar models for each taxonomic group, but with different random effect structures. For amphibians, birds, reef fishes, and marine and terrestrial mammals, we included family nested within order as random effects. For reptiles, we only included family as a random effect, as the dataset is restricted to the order Squamata. For palms, we ran a Linear model without random effects. SE = standard deviation, df = degrees of freedom, *t* = *t* value, *p* = *p*-value, R^2m = marginal R^2, R^2c = conditional R^2.

Model		Estimate	SE	df	*t*	*p*	R^2m	R^2c
**All taxa**	Intercept	0.16	0.14	6.75	1.14	0.29		
Age + (1 | Groups/Order/Family)	**Age**	**0.17**	**0.01**	**25810.00**	**25.31**	**0.00**	0.02	0.52
**Amphibians**	Intercept	0.03	0.03	48.62	0.87	0.39		
Age + (1 | Order/Family)	**Age**	**0.15**	**0.02**	**4178.00**	**9.44**	**0.00**	0.02	0.16
**Birds**	Intercept	0.09	0.03	26.09	2.64	0.01		
Age + (1 | Order/Family)	**Age**	**0.15**	**0.01**	**8477.00**	**13.55**	**0.00**	0.02	0.20
**Reef fishes**	Intercept	0.06	0.03	32.00	1.88	0.07		
Age + (1 | Order/Family)	**Age**	**0.14**	**0.03**	**1427.00**	**5.24**	**0.00**	0.02	0.15
**Terrestrial mammals**	Intercept	0.05	0.05	20.86	1.07	0.30		
Age + (1 | Order/Family)	**Age**	**0.18**	**0.01**	**5128.00**	**12.93**	**0.00**	0.03	0.26
**Marine mammals**	Intercept	-0.08	0.11	7.17	-0.68	0.52		
Age + (1 | Order/Family)	Age	0.10	0.13	69.99	0.77	0.45	0.01	0.25
**Palms**	Intercept	4.64	0.03		157.71	0.00		
Age	**Age**	**0.40**	**0.06**		**6.86**	**0.00**	0.04	0.04

Supplementary Table 5

Insularity modulates the relationship between evolutionary age and range size. Summary of the Linear Mixed Model testing the effect of insularity on the relationship between evolutionary age and range size for all taxa combined (*n* = 25,632). We included families nested within orders, which in turn are nested within taxonomic groups, and the regions (continents or marine regions) as random effects. Additionally, we ran similar models for each taxonomic group, but with different random effect structures. For amphibians, birds, reef fishes, and marine and terrestrial mammals we ran Linear Mixed Models with family nested within order as random effects. We only included family as a random effect for reptiles, as this only includes the order Squamata. We ran a Linear model without random effects for palms, which includes a single family (Arecaceae). SE = standard deviation, df = degrees of freedom, *t* = *t* value, *p* = *p*-value, R^2m = marginal R^2, R^2c = conditional R^2.

Model		Estimate	SE	df	*t*	*p*	R^2m	R^2c
**All taxa**	Intercept	0.06	0.12	6.14	0.54	0.611		
Age x Island + (1| Groups/Order/Family) + (1| Region)	**Age**	**0.13**	**0.01**	**24300.00**	**22.55**	**0.000**		
	**Island**	**-0.44**	**0.01**	**25240.00**	**-57.40**	**0.000**		
	**Age x Island**	**0.15**	**0.01**	**25350.00**	**10.80**	**0.000**	0.11	0.51
**Amphibians**	Intercept	0.06	0.10	5.71	0.66	0.537		
Age x Island + (1| Order/Family) + (1| Region)	**Age**	**0.13**	**0.01**	**4172.60**	**8.38**	**0.000**		
	**Island**	**-0.43**	**0.03**	**4168.90**	**-15.80**	**0.000**		
	Age x Island	-0.05	0.05	4164.62	-1.01	0.312	0.07	0.32
**Birds**	Intercept	0.12	0.08	5.01	1.59	0.174		
Age x Island + (1| Order/Family) + (1| Region)	**Age**	**0.10**	**0.01**	**7715.00**	**10.63**	**0.000**		
	**Island**	**-0.95**	**0.02**	**8770.00**	**-55.73**	**0.000**		
	**Age x Island**	**0.09**	**0.04**	**8713.00**	**2.51**	**0.012**	0.27	0.46
**Reptiles**	Intercept	0.11	0.07	6.55	1.61	0.154		
Age x Island + (1| Family) + (1| Region)	**Age**	**0.14**	**0.01**	**4715.39**	**9.79**	**0.000**		

	**Island**	**-0.21**	**0.02**	**4807.00**	**-13.12**	**0.000**		
	Age x Island	-0.01	0.03	4826.40	-0.44	0.659	0.05	0.25
**Reef fishes**	Intercept	0.01	0.13	1.08	0.07	0.954		
Age x Island + (1| Order/Family) + (1| Region)	**Age**	**0.11**	**0.02**	**1368.00**	**5.29**	**0.000**		
	**Island**	**-0.73**	**0.03**	**1453.00**	**-22.28**	**0.000**		
	**Age x Island**	**0.21**	**0.07**	**1441.00**	**3.19**	**0.001**	0.25	0.46
**Palms**	Intercept	-0.07	0.07	3.38	-0.98	0.394		
Age x Island + (1| Region)	**Age**	**0.18**	**0.03**	**1193.07**	**6.61**	**0.000**		
	**Island**	**-0.32**	**0.09**	**1194.01**	**-3.64**	**0.000**		
	**Age x Island**	**-0.40**	**0.17**	**1192.88**	**-2.39**	**0.017**	0.05	0.13
**Terrestrial mammals**	Intercept	0.08	0.05	18.80	1.60	0.127		
Age x Island + (1| Order/Family) + (1| Region)	**Age**	**0.15**	**0.01**	**4986.03**	**11.30**	**0.000**		
	**Island**	**-0.52**	**0.02**	**4012.40**	**-29.38**	**0.000**		
	**Age x Island**	**0.10**	**0.03**	**5062.43**	**3.20**	**0.001**	0.20	0.37

Supplementary Table 6

Dispersal modulates the relationship between evolutionary age range and size. Summary of the Linear Mixed Model for the relationship between evolutionary age and range size for all taxa (*n* = 25,358), birds (*n* = 8,785), terrestrial mammals (*n* = 4,920), reef fishes (*n* = 1,479), marine mammals (*n* = 70), amphibians (*n* = 4,190), reptiles (*n* = 4,827), and palms (*n* = 1,115). Geographical context (restricted or not restricted to islands) and continents (Americas, Africa, Asia, Australia, Europe) or marine regions for reef fish (Greater Caribbean, Tropical Eastern Pacific) were included as random intercepts, except for marine mammals, which are all restricted to the open ocean and have global, circumtropical or circumtemperate distributions. Family nested in order was included as a random effect. All dispersal-related traits were rescaled for the analyses. SE = standard deviation, df = degrees of freedom, *t* = *t* value, *p* = *p*-value, R^2m = marginal R^2, R^2c = conditional R^2.

Model		Estimate	SE	df	*t*	*p*	R^2m	R^2c
**All taxa**	Intercept	-0.12	0.21	3.89	-0.58	0.592		
Ages x Dispersal + (1|Groups/Order/Family) + (1|Island) + (1|Region)	**Age**	**0.12**	**0.01**	**23970.00**	**21.06**	**0.000**		
	**Dispersal**	**0.14**	**0.01**	**4710.00**	**19.35**	**0.000**		
	**Age x Dispersal**	**-0.03**	**0.01**	**21590.00**	**-3.01**	**0.003**	0.03	0.58
**Birds**	Intercept	-0.36	0.34	2.17	-1.04	0.399		
Age + HWI + (1|Order/Family) + (1|Island) + (1|Region)	**Age**	**0.11**	**0.01**	**7564.00**	**11.92**	**0.000**		
	**HWI**	**0.33**	**0.02**	**2216.00**	**18.27**	**0.000**	0.07	0.68
**Terrestrial Mammals**	Intercept	-0.11	0.20	2.26	-0.54	0.639		
Ages + Body size + (1|Region) + (1|Order/Family) + (1|Island)	**Age**	**0.13**	**0.01**	**4765.72**	**10.58**	**0.000**		
	**Body size**	**0.11**	**0.03**	**666.96**	**4.08**	**0.000**	0.02	0.42
**Reef fishes**	Intercept	-0.29	0.30	2.65	-0.97	0.411		
Age x Body size + Egg type x Body size + (1|Order/Family) + (1|Island) + (1|Region)	**Age**	**0.11**	**0.02**	**1163.14**	**5.56**	**0.000**		
	**Body size**	**0.22**	**0.03**	**101.82**	**7.53**	**0.000**		
	Egg type	0.05	0.03	207.29	1.37	0.172		
	**Age x Body size**	**-0.20**	**0.04**	**1337.90**	**-4.84**	**0.000**		
	**Body size x Egg type**	**-0.17**	**0.06**	**543.00**	**-3.01**	**0.003**	0.06	0.61

**Marine Mammals**	Intercept	-0.11	0.10	4.89	-1.11	0.317		
Age + Body size + (1|Order/Family)	Age	0.03	0.12	69.79	0.24	0.810		
	**Body size**	**0.48**	**0.16**	**14.08**	**3.04**	**0.009**	0.21	0.36
**Amphibians**	Intercept	-0.37	0.25	4.47	-1.47	0.208		
Age x Body size + (1|Order/Family) + (1|Island) + (1|Region)	**Age**	**0.13**	**0.01**	**4183.09**	**8.98**	**0.000**		
	**Body size**	**0.36**	**0.02**	**3589.18**	**16.32**	**0.000**		
	**Age x Body size**	**-0.07**	**0.03**	**4091.19**	**-2.33**	**0.020**	0.09	0.56
**Reptiles**	Intercept	0.00	0.11	4.31	-0.02	0.985		
Age + Body size + (1|Family) + (1|Region) + (1|Island)	**Age**	**0.14**	**0.01**	**4637.00**	**10.07**	**0.000**		
	**Body size**	**0.21**	**0.02**	**2888.00**	**12.99**	**0.000**	0.06	0.29
**Palms**	Intercept	4.35	0.24	3.117	18.22	0.000		
Age + Fruit size + (1|Region) + (1|Island)	**Age**	**0.26**	**0.06**	**1113.00**	**4.44**	**0.000**		
	**Fruit size**	**0.30**	**0.06**	**1110.00**	**5.03**	**0.000**	0.04	0.17

Supplementary Table 7

Number of generations as a proxy for evolutionary age - To investigate whether the number of generations better reflects the age of species than the number of years, we compiled data on generation times for 4,725 mammal species (Pacifici et al., 2014) and 7,622 bird species (Andermann et al., 2020). We calculated the number of generations (assuming non-overlapping generations) since the species' origin by dividing the species' age by generation time. We ran linear mixed models including fixed factors: species age, dispersal traits (hand-wing index (HWI) for birds and aerial dispersal and body size for mammals) and random effects: family nested within order, Island (restricted or not restricted to islands), and Region (continents for terrestrial species: Americas, Asia, Africa, Europe, Australia, or marine regions for reef fish: Greater Caribbean, Tropical Eastern Pacific). SE = standard deviation, df = degrees of freedom, *t* = *t* value, *p* = *p*-value.

Model		Estimate	SE	df	*t*	*p*
**Bird**						
Number of generations + HWI + (1|Order/Family) + (1|Island) + (1|Region)	Intercept	-0.33	0.34	2.17	-0.95	0.435
	**N. generations**	**0.12**	**0.01**	**6677.17**	**11.90**	**0.000**
	**HWI**	**0.31**	**0.02**	**1992.04**	**16.37**	**0.000**
**Terrrestrial mammals**						
Number of generations x Body size + Aerial + (1|Region) + (1|Order/Family) + (1|Island)	Intercept	-0.05	0.20	2.26	-0.26	0.815
	**N. generations**	**0.17**	**0.02**	**4365.47**	**10.25**	**0.000**
	**Body size**	**0.14**	**0.03**	**875.37**	**5.17**	**0.000**
	**Aerial**	**0.30**	**0.13**	**9.97**	**2.26**	**0.048**
	**N. generations x Body size**	**-0.07**	**0.03**	**3475.09**	**-2.25**	**0.024**

Supplementary Table 8

Species occurring exclusively on islands are, on average, younger than species that do not exclusively live on islands. Summary of the Linear Mixed Models for the relationship testing the effect of insularity on species' age. The model included family, nested within order, nested within taxonomic groups and regions (continents for terrestrial species or marine regions for reef fish) as random effects. SE = standard deviation, df = degrees of freedom, *t* = *t* value, *p* = *p*-value.

Model		Estimate	SE	df	*t*	*p*
**All taxa**	Intercept	0.21	0.10	3.48	2.16	0.107
Island + (1 | Groups/Order/Family) + (1|Region)	**Island**	**-0.09**	**0.01**	**24680.00**	**-11.08**	**0.000**
**Birds**	Intercept	0.43	0.06	25.70	7.16	0.000
Island + (1 | Order/Family) + (1|Region)	**Island**	**-0.15**	**0.02**	**8251.11**	**-8.43**	**0.000**
**Amphibians**	Intercept	0.29	0.14	1.88	2.07	0.182
Island + (1 | Order/Family) + (1|Region)	**Island**	**-0.06**	**0.03**	**4123.11**	**-2.22**	**0.027**
**Reptiles**	Intercept	0.19	0.07	28.27	2.68	0.012
Island + (1 | Family) + (1|Region)	Island	-0.03	0.02	4817.54	-1.79	0.074
**Terrestrial mammals**	Intercept	0.39	0.08	21.38	4.61	0.000
Island + (1 | Order/Family) + (1|Region)	**Island**	**-0.16**	**0.02**	**4412.36**	**-8.24**	**0.000**
**Palms**	Intercept	-0.01	0.03	3.16	-0.32	0.767
Island + (1|Region)	**Island**	**-0.25**	**0.08**	**1162.45**	**-3.08**	**0.002**
**Reef fishes**	Intercept	0.06	0.04	18.16	1.49	0.154
Island + (1 | Order/Family) + (1|Region)	**Island**	**-0.12**	**0.04**	**1416.64**	**-3.10**	**0.002**

Supplementary Figure 1

Overview of species ranges size (n = 27,145) and evolutionary age (n = 26,346) for amphibians, birds, reef fish, terrestrial mammals, marine mammals, palms and squamates. Myr = million years.

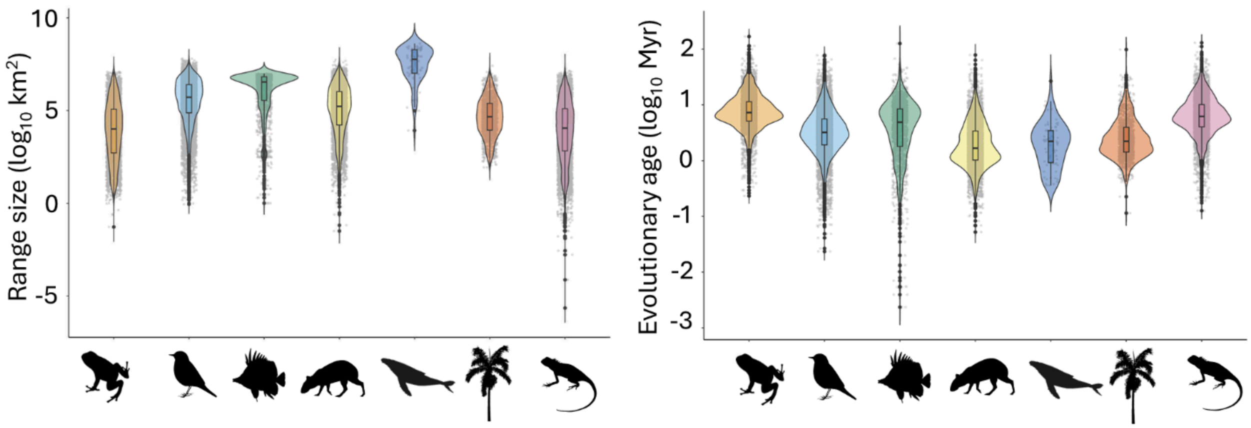

Supplementary Figure 2

The age-range size relationship is robust to outliers. To examine whether the relationship between evolutionary age and range size is affected by extreme values (e.g., very old lineages), we ran a linear mixed model excluding species with evolutionary age values deviating more than three standard deviations from the mean. We included random slopes for each taxonomic group. We used the function 'lmer' from the 'lme4' R package (Bates et al. 2015). We found that the relationship between species' evolutionary age and range size is robust to outliers. The solid line representing the predicted mean (model fit) and the shaded area representing the 95% confidence interval of the predictions, for A) all species, and B-H) separated for the seven broad taxonomic groups: B) amphibians, C) reef fishes, D) birds, E) reptiles, F) palms, G) terrestrial mammals. The dashed line denotes a non-significant relationship for H) marine mammals. See Supplementary Table 3 for summary statistics. n = number of species included. Myr = million years.

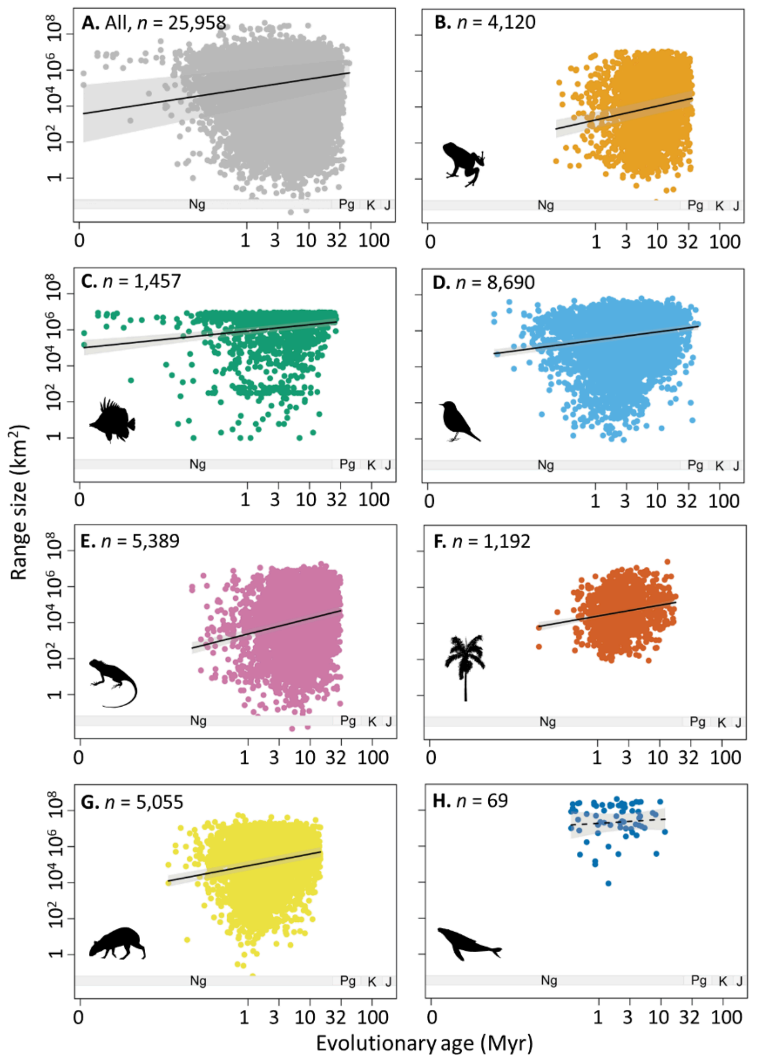

Supplementary Figure 3

Relationships between species adjusted evolutionary age (median age across 100 phylogenetic trees) and range size for a) all species, and b-h) separated for the six taxonomic groups: b) amphibians, c) reef fishes, d) birds, e) reptiles, f) palms, g) terrestrial mammals, and h) marine mammals. The black lines denote a significant relationship between evolutionary age and range size, whereas the dashed line denotes a non-significant relationship. Geological periods are denoted as Ng = Neogene, Pg = Paleogene, K = Cretaceous, and J = Jurassic. Data were log_{10}-transformed for analysis and plotting. n = number of species. Myr = million years. See Supplementary Table 4 for more details on the method.

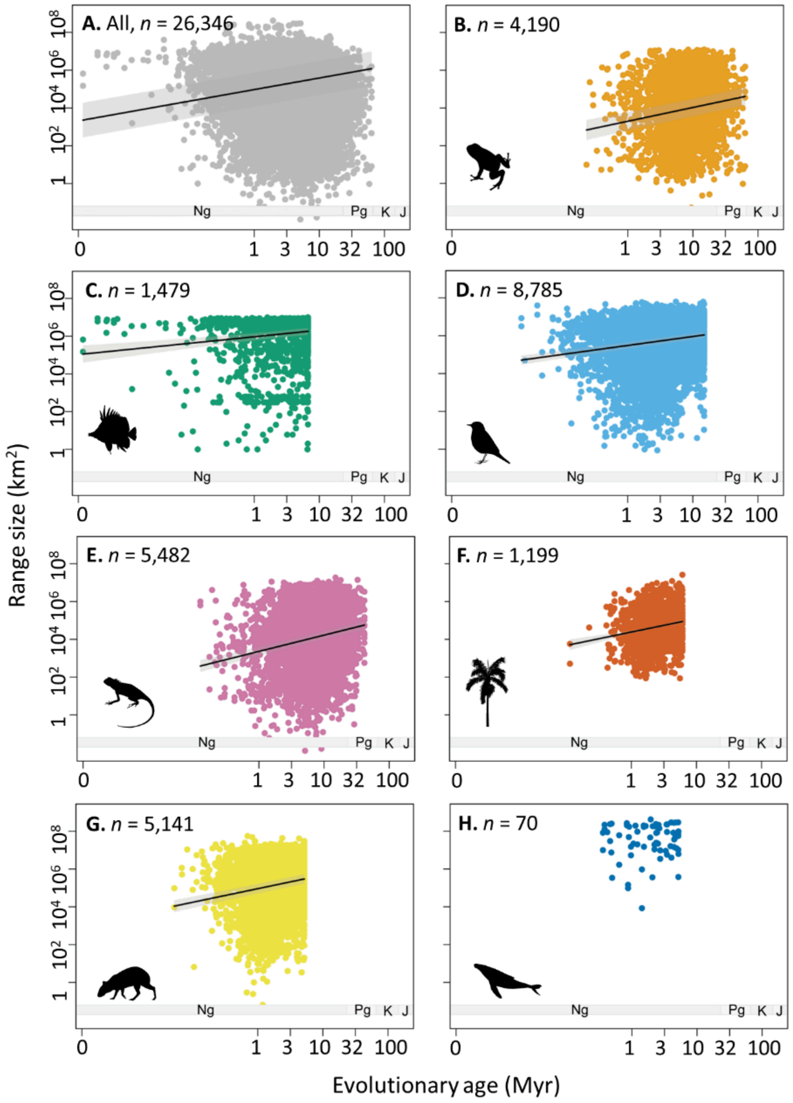

Supplementary Figure 4

Range size distribution for species restricted to islands vs. not restricted to islands. Overall, species restricted to islands have, on average, smaller range sizes than species not restricted to islands (Estimate = -0.47, SE = 0.007, $t(25{,}220) = -59{,}26$, $p < 0.001$, $n = 25{,}632$, $R^2m = 0.19$, $R^2c = 0.47$). This is also the case for each taxonomic group: Terrestrial mammals (Estimate = -0.55, SE = 0.02, $t(4{,}094.08) = -31.04$, $p < 0.001$, $n = 5{,}142$, $R^2m = 0.18$, $R^2c = 0.35$), reef fishes (Estimate = -0.77, SE = 0.03, $t(1{,}451) = -24.21$, $p < 0.001$, $n = 1{,}479$, $R^2m = 0.24$, $R^2c = 0.45$), palms (Estimate = -0.26, SE = 0.08, $t(1{,}196.62) = -3.32$, $p < 0.001$, $n = 1{,}199$, $R^2m = 0.009$, $R^2c = 0.09$), birds (Estimate = -0.97, SE = 0.02, $t(8{,}717.08) = -59{,}71$, $p < 0.001$, $n = 8{,}785$, $R^2m = 0.26$, $R^2c = 0.45$), reptiles (Estimate = -0.21, SE = 0.02, $t(4{,}809.02) = -13.34$, $p < 0.001$, $n = 4{,}837$, $R^2m = 0.03$, $R^2c = 0.24$), and amphibians (Estimate = -0.44, SE = 0.03, $t(4{,}172.5) = -16.12$, $p < 0.001$, $n = 4{,}190$, $R^2m = 0.06$, $R^2c = 0.31$).

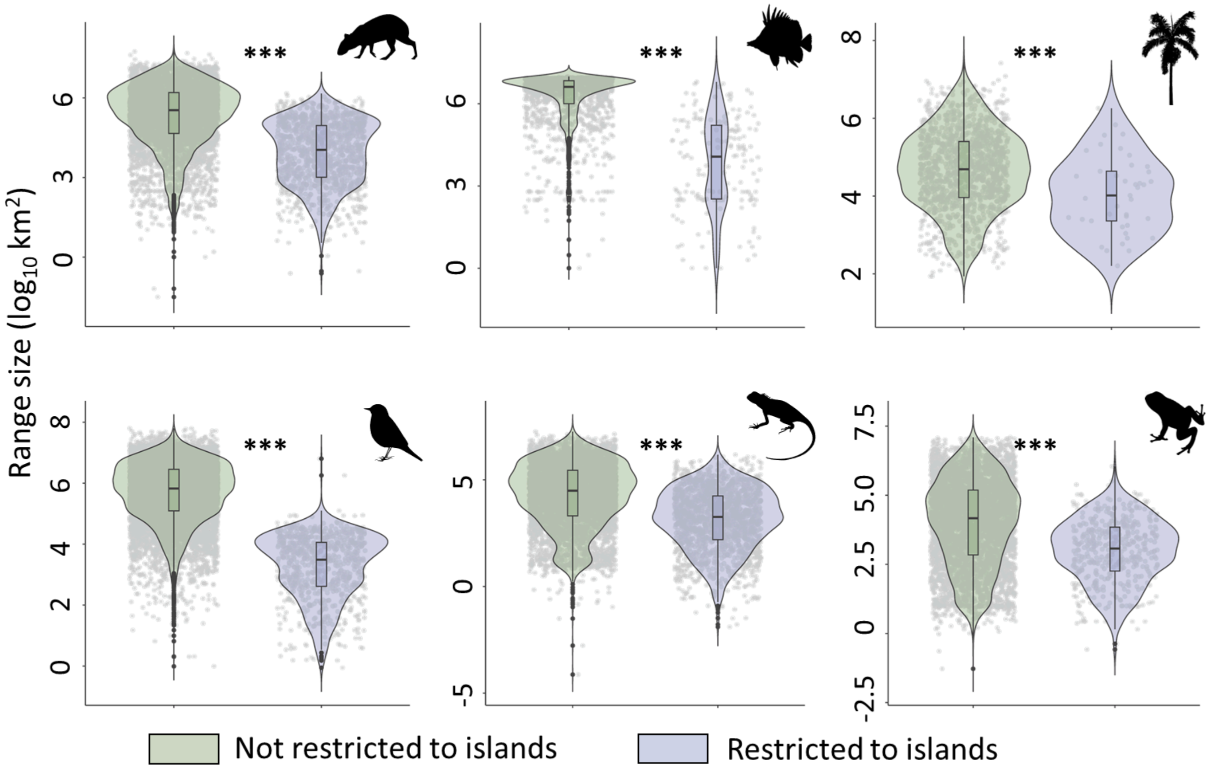

Supplementary Figure 5

Evolutionary age distribution for species restricted to islands vs. not restricted to islands. We ran a linear mixed model with island as a fixed effect, and family nested within order nested within taxonomic group as random effects. Species restricted to islands are, on average, younger than species not restricted to islands (Estimate = -0.09, SE = 0.001, t = -11.08, $p < 0.0001$, n = 25,632). Summary statistics for the model, including all taxa and individual models for each taxonomic group, are presented in Supplementary Table 8. Myr = million years.

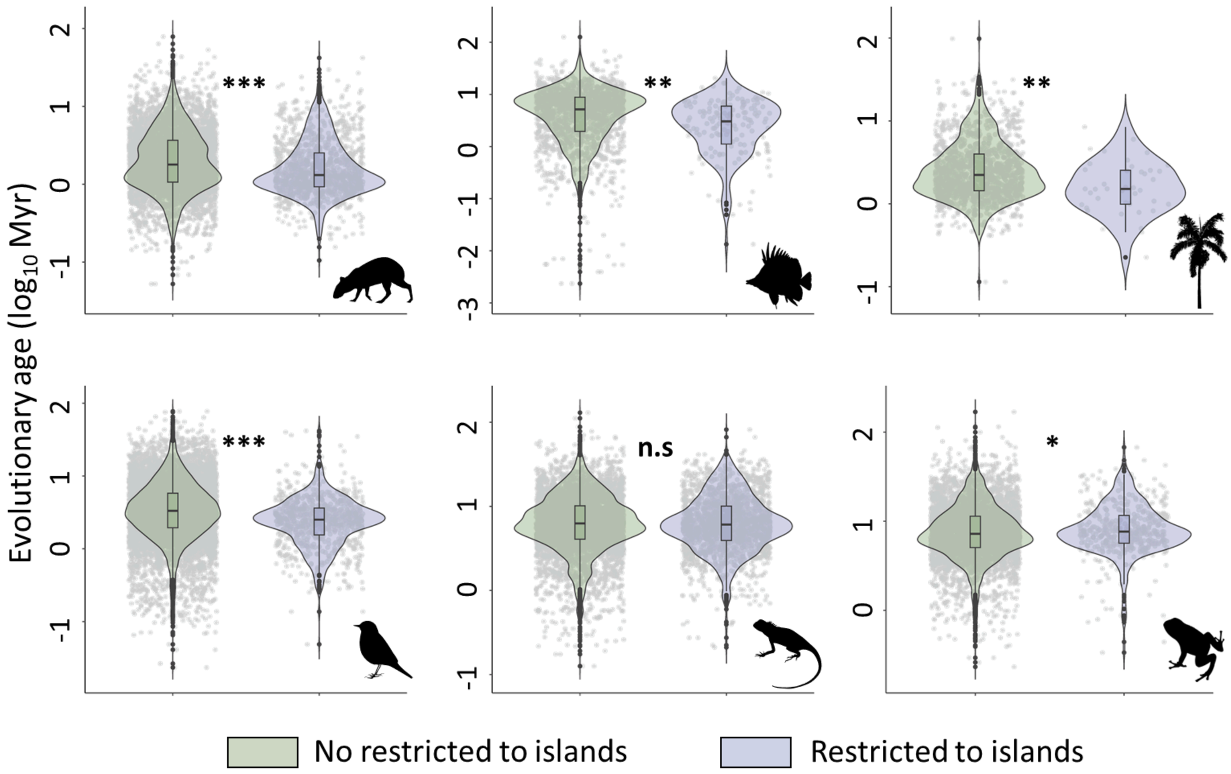

Supplementary Figure 6

We examined the effect of sample size on the relationship between species age and range size by randomly sampling species from the full dataset (*n* = 26,345). We ran linear mixed-effects models for each of the 1,000 randomizations. The models included ‘family’ nested within ‘order’, which was nested within the ‘broad taxonomic group’ as random effects. The results indicate that the effect of age becomes increasingly uncertain with smaller sample sizes. For samples with fewer than 200 species, the effect can shift to negative, with the 95% confidence interval (CI) crossing 0. Significance also decreases with smaller sample sizes, with a high proportion of models showing non-significant effects for samples with fewer than 500 species, and the median effect becomes non-significant for samples with fewer than 200 species. Red whiskers represent the 95% data range, while the black horizontal line within the barplot indicates the median.

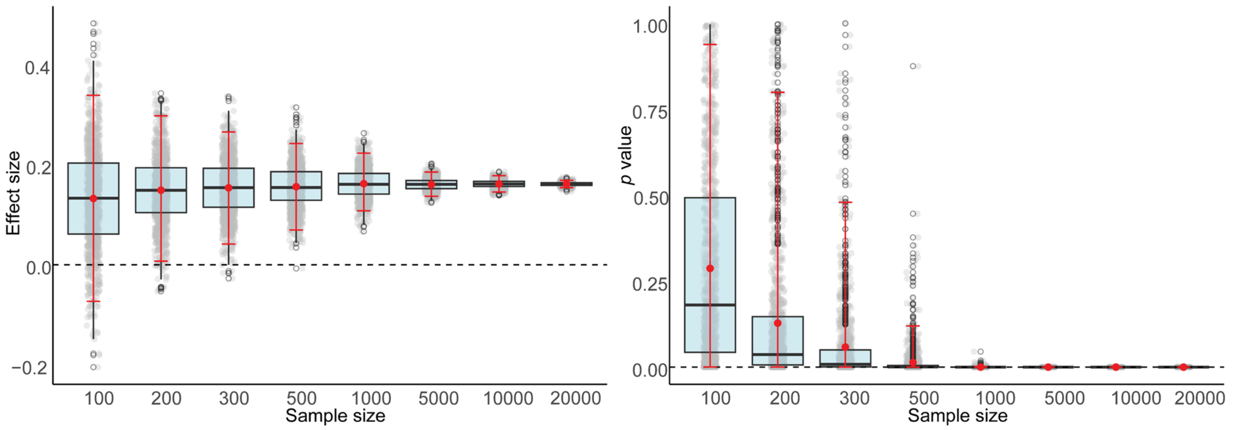

References

1. Abellán, P., Ribera, I. Geographic location and phylogeny are the main determinants of the size of the geographical range in aquatic beetles. BMC Evol Biol 11, 344 (2011). https://doi.org/10.1186/1471-2148-11-344

2. Andermann, T., Faurby, S., Cooke, R., Silvestro, D., & Antonelli, A. (2021). Iucn_sim: A new program to simulate future extinctions based on IUCN threat status. *Ecography*, *44*(2), 162-176. https://doi.org/10.1111/ecog.05110

3. Böhm, M., Kemp, R., Williams, R., Davidson, A. D., Garcia, A., McMillan, K. M., Bramhall, H. R., & Collen, B. (2017). Rapoport's rule and determinants of species range size in snakes. *Diversity and Distributions*, *23*(12), 1472-1481. https://doi.org/10.1111/ddi.12632

4. Böhning-Gaese, K., Caprano, T., van Ewijk, K. & Veith, M. (2006). Range size: disentangling current traits and phylogenetic and biogeographic factors. The American Naturalist., 167(4), 555–567.

5. Etienne R, Haegeman B (2023). DDD: Diversity-Dependent Diversification. R package version 5.2.2, <https://CRAN.R-project.org/package=DDD>.

6. Foote, M. (2007). Symmetric waxing and waning of marine invertebrate genera. Paleobiology, 33(4), 517-529. doi:10.1666/06084.1

7. Gaston, K.J. & Blackburn, T.M. (1997). Age, area and avian diversification. Biological Journal of the Linnean Society. 62: 239-253.

8. Guo, Q., Qian, H., Zhang, J., & Liu, P. (2024). The relationships between species age and range size. *Journal of Biogeography*, *51*(6), 1095-1103. https://doi.org/10.1111/jbi.14809

9. Hodge, J. and Bellwood, D.R. (2015), Species age and geographical range. Global Ecology and Biogeography, 24: 495-505. https://doi.org/10.1111/geb.12264

10. Jablonski, D. 1987. Heritability at the species level: analysis of geographic ranges of Cretaceous mollusks. Science. 238:360–363.

11. Jones, K., Sechrest, W., & Gittleman, J. (2005). Age and area revisited: Identifying global patterns and implications for conservation. In A. Purvis, J. Gittleman, & T. Brooks (Eds.), Phylogeny and Conservation (Conservation Biology, pp. 141-165). Cambridge: Cambridge University Press. doi:10.1017/CBO9780511614927.007

12. Leão, T.C.C., Lughadha, E.N. and Reich, P.B. (2020), Evolutionary patterns in the geographic range size of Atlantic Forest plants. Ecography, 43: 1510-1520. https://doi.org/10.1111/ecog.05160

13. Miller, A.I. 1997. A new look at age and area: the geographic and environmental expansion of genera during the Ordovician Radiation. Paleobiology. 23(4):410-419.

14. Mora, C., Treml, E., Robert, J., Crosby, K., Roy, D. & Tittensor, D.P. (2012) High connectivity among habitats precludes the relationship between dispersal and range size in tropical reef fishes. Ecography, 35(1), 89– 96.

15. Raia P, Meloro C, Loy A, Barbera C. Species occupancy and its course in the past: macroecological patterns in extinct communities. Evol. Ecol. Res. 2006;8:181–194.

16. Ricklefs, R. E., & Latham, R. E. (1992). Intercontinental Correlation of Geographical Ranges Suggests Stasis in Ecological Traits of Relict Genera of Temperate Perennial Herbs. The American Naturalist. https://doi.org/10.1086/285388

17. Schurr, F.M., Midgley, G.F., Rebelo, A.G., Reeves, G., Poschlod, P. & Higgins, S.I. (2007) Colonization and persistence ability explain the extent to which plant species fill their potential range. Global Ecology and Biogeography., 16, 449–459.

18. Swaegers, J., Janssens, S. B., Ferreira, S., Watts, P. C., Mergeay, J., McPeek, M. A., & Stoks, R. (2014). Ecological and evolutionary drivers of range size in Coenagrion damselflies. *Journal of Evolutionary Biology*, *27*(11), 2386-2395. https://doi.org/10.1111/jeb.12481

19. Taylor, C. M., & Gotelli, N. J. (1994). The Macroecology of Cyprinella: Correlates of Phylogeny, Body Size, and Geographical Range. *The American Naturalist*. https://doi.org/10.1086/285694

20. Pacifici, Michela et al. (2014). Data from: Generation length for mammals [Dataset]. Dryad. https://doi.org/10.5061/dryad.gd0m3

21. Paul, J.R. & Tonsor, S.J. 2008. Explaining Geographic Range Size by Species Age: A Test Using Neotropical Piper Species. In: Carson, W & Schnitzer, S (eds.). Tropical Forest Community Ecology.Wiley-Blackwell. 536 p.

22. Paul, J.R., Morton, C., Taylor, C.M., and Tonsor, S.J. (2009) Evolutionary Time for Dispersal Limits the Extent but Not the Occupancy of Species' Potential Ranges in the Tropical Plant Genus Psychotria (Rubiaceae). The American Naturalist. 173(2): 188-199

23. Pepke, M. L., Irestedt, M., Fjeldså, J., Rahbek, C., & Jønsson, K. A. (2019). Reconciling supertramps, great speciators and relict species with the taxon cycle stages of a large island radiation (Aves: Campephagidae). Journal of Biogeography, 46(6), 1214-1225. https://doi.org/10.1111/jbi.13577

24. Price, T.D., Helbig, A.J. & Richman, A.D. 1997. Evolution of breeding distributions in the old world leaf warblers (genus Phylloscopus). Evolution. 51(2): 552-561.

25. Webb, T.J. & Gaston, K.J. (2000) Geographic range size and evolutionary age in birds. Proceedings of the Royal Society B, 267(1455), 1843– 1850.

26. Weber, M., Stevens, R. D., Lorini, M. L., & V. Grelle, C. E. (2014). Have old species reached most environmentally suitable areas? A case study with South American

phyllostomid bats. Global Ecology and Biogeography, 23(11), 1177-1185. https://doi.org/10.1111/geb.12198

27. Wollenberg, K.C., Vieites, D.R., Glaw, F. & Vences, M. (2011) Speciation in little: the role of range and body size in the diversification of Malagasy mantellid frogs. BCM Evolutionary Biology. 11, 217.
