# Supplementary material for: Evolutionary age correlates with range size across plants and animals
Source: Nat Commun. 2025 Aug 23;16:7894. doi: 10.1038/s41467-025-62124-y (PMC12375016; doi:10.1038/s41467-025-62124-y)
Supplement: Supplementary file 2 — Description of Addtional Supplementary Files [file 41467_2025_62124_MOESM2_ESM.pdf]

Description of Additional Supplementary Files

Supplementary Data 1: Results of individual analyses of age-range size relationships per major taxonomic group (birds, mammals, reptiles, amphibians, reef fishes, and palms), and at order and family scales.

Supplementary Data 2: Representative species selected for each order or family used in phylogenetic reconstructions.
