# Supplementary material for: Evolutionary age correlates with range size across plants and animals
Source: Nat Commun. 2025 Aug 23;16:7894. doi: 10.1038/s41467-025-62124-y (PMC12375016; doi:10.1038/s41467-025-62124-y)
Supplement: Supplementary file 5 — Reporting Summary [file 41467_2025_62124_MOESM5_ESM.pdf]

nature portfolio

Corresponding author(s): Adriana Alzate

Last updated by author(s): Dec 14, 2024

Reporting Summary

Nature Portfolio wishes to improve the reproducibility of the work that we publish. This form provides structure for consistency and transparency in reporting. For further information on Nature Portfolio policies, see our Editorial Policies and the Editorial Policy Checklist.

Statistics

For all statistical analyses, confirm that the following items are present in the figure legend, table legend, main text, or Methods section.

n/a	Confirmed	
☐	☒	The exact sample size (*n*) for each experimental group/condition, given as a discrete number and unit of measurement
☒	☐	A statement on whether measurements were taken from distinct samples or whether the same sample was measured repeatedly
☐	☒	The statistical test(s) used AND whether they are one- or two-sided<br>*Only common tests should be described solely by name; describe more complex techniques in the Methods section.*
☐	☒	A description of all covariates tested
☐	☒	A description of any assumptions or corrections, such as tests of normality and adjustment for multiple comparisons
☐	☒	A full description of the statistical parameters including central tendency (e.g. means) or other basic estimates (e.g. regression coefficient) AND variation (e.g. standard deviation) or associated estimates of uncertainty (e.g. confidence intervals)
☐	☒	For null hypothesis testing, the test statistic (e.g. *F*, *t*, *r*) with confidence intervals, effect sizes, degrees of freedom and *P* value noted<br>*Give P values as exact values whenever suitable.*
☒	☐	For Bayesian analysis, information on the choice of priors and Markov chain Monte Carlo settings
☒	☐	For hierarchical and complex designs, identification of the appropriate level for tests and full reporting of outcomes
☐	☒	Estimates of effect sizes (e.g. Cohen's *d*, Pearson's *r*), indicating how they were calculated

Our web collection on statistics for biologists contains articles on many of the points above.

Software and code

Policy information about availability of computer code

Data collection

We collated range size data for 27,145 species (Supplementary Figure 1). We obtained distribution maps from the International Union for Conservation of Nature's Red List of Threatened Species 65 (IUCN) for amphibians, marine and terrestrial mammals, and reptiles. For birds, we obtained range size data from the AVONET database 66 and for palms from Hill et al. 67. For reef-associated bony fishes (class 'Actinopterygii'), we obtained occurrence data from Robertson & van Tassell 68 and Robertson & Allen 69. We documented and/or approximated range size as the extent of occurrence (EOO) within a projection that accounts for the curvature of the Earth. We chose EOO to capture the range extent, as EOO should be directly affected by time for range expansion while minimizing bias in estimates of natural (i.e. pre-human) range sizes due to local extinction, habitat fragmentation, and other factors (e.g. ecological niche) that influence range size independent from the expansion process.
We collated data on the evolutionary age of 26,346 species (Supplementary Figure 1). We obtained 100 phylogenetic trees for birds 70, squamates 71, amphibians 72 and mammals 73 from 'Vertlife.org'. We obtained 100 phylogenetic trees for fish from the 'Fish Tree of Life' 74, and for palms (Arecaceae) from Faurby et al. 75. We estimated the overall age per species as the median branch length of the terminal nodes (i.e., species or tips) from 100 phylogenetic trees.
We collated data on species dispersal abilities for 26,474 species. For mammals, we obtained data on body size (continuous: body mass in g) and flight ability (categorical: yes = 1, no = 0) from Phylacine 1.2.1 76,77. For birds, we obtained hand-wing index (HWI, continuous) data from AVONET 66. For amphibians, we obtained body size data (continuous: snout-vent length in Anura, and total length in Gymnophiona and Caudata in mm) from AmphiBIO 78 and for reptiles (maximum length in mm) from Feldman et al. 79 and Meiri 80. We obtained body size data (maximum length in cm) for reef fishes from Alzate et al. 81, Robertson & van Tassell 68and Robertson & Allen 69. For palms, we obtained fruit size (average fruit length in mm) data from Kissling et al. 82.
We collated data on insularity (land masses smaller than Australia) for 26,784 species. We classified species as "restricted to islands" if found exclusively on islands. Conversely, species "not restricted to islands" are those found on continents or on both continents and islands. Despite some island endemic species inhabiting multiple islands, their maximum potential range is still generally less than that of continental species

or species that live both on continents and islands. We obtained data on island endemicity from Phylacine 1.2.1 76,77 for terrestrial mammals. We considered bird species as "restricted to islands" when they were reported to be 100% associated with islands in Sheard et al. 83. For amphibians, we obtained information on insularity by overlaying species distribution maps with a shapefile of islands of the world, which is based on a digital elevation model at 30 m of pixel resolution. For reptiles, we obtained information on geographical context ("restricted to islands" vs "not restricted to islands") from Meiri 80, and for reef fishes from Alzate el. al. 81, Robertson & van Tassell 68and Robertson & Allen 69. We classified palm species as restricted or not restricted to islands following Cassia-Silva et al. 84.
We collated data on generation times for 4,725 mammal species from Pacifici et al. 2014 85and for 7,499 bird species from Andermann et al. 2020 86.
See Expanded Methods for a more extensive description.

Data analysis

To examine the overall effect of age on range size, we ran a linear mixed model including 26,346 species from seven broad taxonomic groups (amphibians, reef fishes, birds, reptiles, palms, terrestrial and marine mammals). We used the function 'lmer' from the 'lme4' R package 87. To account for phylogenetic structure, we included 'family' nested within 'order' nested within 'broad taxonomic group' as a random effect. We also ran individual models for each taxonomic group with the same random effect structure (family nested within order), except for reptiles for which only family was included, and palms for which no random effects were included.
We tested whether the effect of species age on range size is expected by chance by building null models in which we randomized the median species' age 1,000 times. We ran these null models for the complete set of species and each broad taxonomic group. We built a linear mixed-effects model for each randomisation. The random effect structure was the same as for the main model. We considered the effect of age on range size to be expected by chance if the observed effect size falls within the distribution of the 1,000 effect size from the null models.
To examine how the age-range size relationship varied among taxonomic groups, we ran linear models at three taxonomic levels: at the broad taxonomic level (reef fishes, birds, terrestrial mammals, marine mammals, amphibians, squamates and palms), at the order level (88 orders), and the family level (418 families). We performed a meta-analysis for each taxonomic level to test the overall effect of age on range size based on all individual relationships. We fitted a random effects model, including the Z-score values with their corresponding standard errors, using the function 'rma' from the R package 'metafor' 88.
We tested the effect of insularity on the relationship between age and range size by running a linear mixed model with species age and insularity as additive and interacting fixed effects and 'family' nested within 'order' nested within 'broad taxonomic group', and continents (Americas, Africa, Asia, Australia, Europe) or marine regions for reef fishes (Greater Caribbean, Tropical Eastern Pacific) as random effects. We also ran individual models for each taxonomic group, but with different random effect structures. We included continent, and 'family' nested within 'order' for all groups, except for reptiles for which we included continent and 'family' as random effects, and palms for which we only included continent as random effect. Marine mammals were excluded from the analysis as too few species were restricted to islands. We used the function 'lmer' from the 'lme4' R package 87.
To test whether the relationship between age and range size depended on dispersal, we ran linear mixed-effects models for the six broad taxonomic groups (amphibians, reef fishes, birds, reptiles, palms, terrestrial and marine mammals). We included age and dispersal-related traits as additive and interactive fixed effects. We accounted for insularity and palaeogeographic history by including whether species are restricted to islands (restricted or not restricted to islands) and continents (Americas, Africa, Asia, Australia, Europe) or marine regions for reef fishes (Greater Caribbean, Tropical Eastern Pacific), as random intercepts, except for marine mammals, which are all restricted to the open ocean and have global, circumtropical or circumtemperate distributions. To account for phylogenetic structure, we included 'family' nested within 'order' as random slopes for birds, terrestrial and marine mammals, reef fishes and amphibians. For reptiles, we only included 'family' as a random effect, and for palms we did not include taxonomic structure as a random effect.
To test whether the number of generations is a better proxy of species age than the number of years, we investigated whether using different species' age metrics (millions of years vs. number of generations) influences the relationship between species age and range size. To this end, we ran linear mixed models including fixed factors: species age, dispersal traits (Hand-wing Index for birds and aerial dispersal and body size for mammals) and random effects: 'family' nested within 'order', Island, and Region. We calculated the number of generations (assuming non-overlapping generations) since the species' origin by dividing the species' age by generation time.
To meet linearity assumptions, dispersal-related traits, age and range size were log-transformed (log10) and dispersal-related traits were rescaled using the function 'rescale' from the 'arm' R package 89. All models were standardized using the function 'standardize' from the 'arm' R package 89.

For manuscripts utilizing custom algorithms or software that are central to the research but not yet described in published literature, software must be made available to editors and reviewers. We strongly encourage code deposition in a community repository (e.g. GitHub). See the Nature Portfolio guidelines for submitting code & software for further information.

Data

Policy information about availability of data

All manuscripts must include a data availability statement. This statement should provide the following information, where applicable:

- Accession codes, unique identifiers, or web links for publicly available datasets
- A description of any restrictions on data availability
- For clinical datasets or third party data, please ensure that the statement adheres to our policy

The datasets generated during the current study are available in Dryad.

Research involving human participants, their data, or biological material

Policy information about studies with human participants or human data. See also policy information about sex, gender (identity/presentation), and sexual orientation and race, ethnicity and racism.

Reporting on sex and gender: no applicable

Reporting on race, ethnicity, or: no applicable

other socially relevant groupings	
Population characteristics	no applicable
Recruitment	no applicable
Ethics oversight	no applicable

Note that full information on the approval of the study protocol must also be provided in the manuscript.

Field-specific reporting

Please select the one below that is the best fit for your research. If you are not sure, read the appropriate sections before making your selection.

☐ Life sciences ☐ Behavioural & social sciences ☒ Ecological, evolutionary & environmental sciences

For a reference copy of the document with all sections, see nature.com/documents/nr-reporting-summary-flat.pdf

Ecological, evolutionary & environmental sciences study design

All studies must disclose on these points even when the disclosure is negative.

Study description

More than 40 thousand species of plants and animals are facing extinction worldwide. Range size is one of the strongest determinants of extinction risk, but the causes underlying the wide variation in natural range sizes remain poorly understood. Here, we investigate how species' age is related to present-day range size for over 26,000 species of mammals, birds, reptiles, amphibians, reef fishes, and plants. We show that, on average, older species have larger ranges across all groups except for marine mammals, but the strength of the age-range size relationship depends on taxonomic scale. Furthermore, while our results confirm the well-established pattern of smaller range sizes for species restricted to islands (compared to mainland) or with limited dispersal abilities (compared to good dispersers), we show that the correlation between species age and range size is stronger in these groups, suggesting that island dynamics and dispersal ability modulate this relationship. Our study reveals that species with small ranges, and thus increased extinction risk, tend to be restricted to islands, are poor dispersers, or have recently evolved.

Research sample

26,345 species with available range size and evolutionary age information for seven taxonomic groups: birds, amphibians, scaled reptiles (Squamata), palms, reef fishes from the Greater Caribbean and the Tropical Eastern Pacific, and marine and terrestrial mammals.

Sampling strategy

The sample size was determined by the available data on species geographical distributions (27,145 species), and evolutionary age (26,346).

Data collection

We collated range size data for 27,145 species (Supplementary Figure 1). We obtained distribution maps from the International Union for Conservation of Nature's Red List of Threatened Species 65 (IUCN) for amphibians, marine and terrestrial mammals, and reptiles. For birds, we obtained range size data from the AVONET database 66 and for palms from Hill et al. 67. For reef-associated bony fishes (class 'Actinopterygii'), we obtained occurrence data from Robertson & van Tassell 68 and Robertson & Allen 69. We documented and/or approximated range size as the extent of occurrence (EOO) within a projection that accounts for the curvature of the Earth. We chose EOO to capture the range extent, as EOO should be directly affected by time for range expansion while minimizing bias in estimates of natural (i.e. pre-human) range sizes due to local extinction, habitat fragmentation, and other factors (e.g. ecological niche) that influence range size independent from the expansion process.
We collated data on the evolutionary age of 26,346 species (Supplementary Figure 1). We obtained 100 phylogenetic trees for birds 70, squamates 71, amphibians 72 and mammals 73 from 'Vertlife.org'. We obtained 100 phylogenetic trees for fish from the 'Fish Tree of Life' 74, and for palms (Arecaceae) from Faurby et al. 75. We estimated the overall age per species as the median branch length of the terminal nodes (i.e., species or tips) from 100 phylogenetic trees.
We collated data on species dispersal abilities for 26,474 species. For mammals, we obtained data on body size (continuous: body mass in g) and flight ability (categorical: yes = 1, no = 0) from Phylacine 1.2.1 76,77. For birds, we obtained hand-wing index (HWI, continuous) data from AVONET 66. For amphibians, we obtained body size data (continuous: snout-vent length in Anura, and total length in Gymnophiona and Caudata in mm) from AmphiBIO 78 and for reptiles (maximum length in mm) from Feldman et al. 79 and Meiri 80. We obtained body size data (maximum length in cm) for reef fishes from Alzate et al. 81, Robertson & van Tassell 68and Robertson & Allen 69. For palms, we obtained fruit size (average fruit length in mm) data from Kissling et al. 82.
We collated data on insularity (land masses smaller than Australia) for 26,784 species. We classified species as "restricted to islands" if found exclusively on islands. Conversely, species "not restricted to islands" are those found on continents or on both continents and islands. Despite some island endemic species inhabiting multiple islands, their maximum potential range is still generally less than that of continental species or species that live both on continents and islands. We obtained data on island endemicity from Phylacine 1.2.1 76,77 for terrestrial mammals. We considered bird species as "restricted to islands" when they were reported to be 100% associated with islands in Sheard et al. 83. For amphibians, we obtained information on insularity by overlaying species distribution maps with a shapefile of islands of the world, which is based on a digital elevation model at 30 m of pixel resolution. For reptiles, we obtained information on geographical context ("restricted to islands" vs "not restricted to islands") from Meiri 80, and for reef fishes from Alzate el. al. 81, Robertson & van Tassell 68and Robertson & Allen 69. We classified palm species as restricted or not restricted to islands following Cassia-Silva et al. 84.
We collated data on generation times for 4,725 mammal species from Pacifici et al. 2014 85and for 7,499 bird species from Andermann et al. 2020 86.
See Expanded Methods for a more extensive description.

Timing and spatial scale	All data was collated between 2020 to 2022.<br>Information on geographical distributions is global for birds, amphibians, scaled reptiles (Squamata), palms, and marine and terrestrial mammals. For reef fishes, geographical distribution were available for all species from the Greater Caribbean and the Tropical Eastern Pacific.
Data exclusions	We excluded data for:<br>Extinct species, Extinct in the Wild species<br>Marine mammals 'occurring on mainland' = 3 species<br>Species without complete data on range sizes and evolutionary age for the main analysis on the relationship between evolutionary age and range size.<br>Species without complete data on range sizes, evolutionary age and insularity for the analyses on the relationship between evolutionary age and range size modulated by geographical constraints.<br>Species without complete data on range sizes, evolutionary age, dispersal-related traits and insularity for the analyses on the relationship between evolutionary age and range size modulated by dispersal.<br>Marine birds, because due to the particular biology they were mainly only occurring on islands but attaining global very broad distributions<br>Families or orders with less than three species when testing the effect of species age on range size on single families or orders<br>Species with artificially small ranges (0.01 km2) due to uncertainty of their distribution for palms.
Reproducibility	Reproducibility is not relevant for our study as we used the most complete and unique information on species ranges, based on the available IUCN distributions maps, and on evolutionary age, based on the only complete phylogenies available.<br>We, however, described in detail the methodological procedure, the data collection procedure and provide the dataset for reproducing our results.
Randomization	There was not randomization necessary in this study, as no experiments were performed. Allocation of data (species) to groups: taxonomic groups, Orders, Families, insularity and continents was based on current taxonomic classifications, on reported insularity information and on geographical information.
Blinding	The nature of our study did not necessitate blinding procedures because we do not performed experiments. Also the experimental design, data collection, and analysis were inherently unaffected by the species involve. In fact for data collection and classification, the species identity is necessary. Consequently, blinding was not applicable to our research methodology.

Did the study involve field work? ☐ Yes ☒ No

Reporting for specific materials, systems and methods

We require information from authors about some types of materials, experimental systems and methods used in many studies. Here, indicate whether each material, system or method listed is relevant to your study. If you are not sure if a list item applies to your research, read the appropriate section before selecting a response.

Materials & experimental systems

n/a	Involved in the study
☒	☐ Antibodies
☒	☐ Eukaryotic cell lines
☒	☐ Palaeontology and archaeology
☒	☐ Animals and other organisms
☒	☐ Clinical data
☒	☐ Dual use research of concern
☒	☐ Plants

Methods

n/a	Involved in the study
☒	☐ ChIP-seq
☒	☐ Flow cytometry
☒	☐ MRI-based neuroimaging

Plants

Seed stocks	no applicable
Novel plant genotypes	no applicable
Authentication	no applicable
